# Influence of the intelligent knee osteoarthritis lifestyle app (iKOALA) on knee joint pain

**DOI:** 10.1186/s12891-024-07198-3

**Published:** 2024-01-24

**Authors:** Richard D.M. Stevenson, Enhad A. Chowdhury, Jesudas Lobo, Max J. Western, James L.J. Bilzon

**Affiliations:** 1 Department for Health, The University of Bath, Bath, BA2 7AY UK; 2 Centre for Sport, Exercise and Osteoarthritis Research Versus Arthritis, Bath, BA2 7AY UK; 3 Department of Computer Science, The University of Bath, Bath, BA2 7AY UK

**Keywords:** Knee osteoarthritis, Musculoskeletal, Physical activity, Mobile application, Digital health

## Abstract

**Background:**

The intelligent knee osteoarthritis lifestyle app (iKOALA) has been co-developed with target users to extend the support for physical activity (PA) and musculoskeletal health, beyond short-term structured rehabilitation, using personalised PA guidance, education, and social support. The purpose of this study was to assess the preliminary effectiveness and usability of the iKOALA digital intervention on indices of musculoskeletal (MSK) health, symptoms, and physical activity levels in a broad range of individuals with knee osteoarthritis (KOA) over 12 weeks to inform the design of a larger randomised controlled trial.

**Methods:**

Thirty-eight (33 female) participants living in the UK with a mean (SD) age of 58 (± 9) years diagnosed radiographically or clinically with KOA completed a 12-week user trial of the iKOALA. Participants completed an in-app physical activity questionnaire which intelligently recommended suitable strengthening and aerobic based activities to individuals. Throughout the trial, participants wore a physical activity monitor and were given access to functions within the app (physical activity (PA) reminders, information and education, symptom and PA tracking as well as social support forums) to support them in maintaining their PA plan. Participants completed a MSK questionnaire for chronic symptoms and quality of life (MSK-HQ) as well as an acute iKOALA symptoms questionnaire (confidence, fatigue, mood, pain during the day/night, sleep and ability to walk) in the week prior to starting and following completion of the trial.

**Results:**

Physical activity levels were consistent over the 12 weeks with total daily steps of 9102 (± 3514) in week 1, 9576 (± 4214) in week 6 and 9596 (± 3694) in week 12. Group mean changes in all iKOALA MSK symptom scores and the total MSK-HQ (pre 33.1 (7.6) vs. post 40.2 (7.6)) score improved significantly (*p* < .001, 95% CI [-8.89, -5.16]) over the 12-week period.

**Conclusions:**

Physical activity levels were maintained at a high level throughout the 12 weeks. Significant improvements in mean MSK symptom scores and the total MSK-HQ score were also observed. Efforts to ensure more generalised reach amongst sex and socioeconomic status of the digital intervention in a randomised controlled clinical trial are warranted.

## Background

The number of people living with a long term health condition continues to rise in many developed countries which is increasing the pressure on health and social care systems [1, 2]. If managed poorly, conditions such as osteoarthritis (OA) can be detrimental to both the individual and wider society through a reduced quality of life, loss of work ability and higher medical costs associated with complications [2]. When managed well, people with long-term conditions can live independent and healthful lives into their later years, reducing the need for regular medical treatment [3, 4].

Empowering patients to self-manage their condition is considered an important step toward successful health management [4, 5] and the use of mobile health (mHealth) apps have emerged as a promising tool to support this approach [6]. Purported benefits of mHealth apps are their ability to: effectively reach a large number of people at relatively low cost [7], reduce clinician contact through remote communication and monitoring [8], and providing instantly accessible and tailored patient education, coaching and social support [9]. Consequently, mHealth applications have been developed for a range of conditions [10–13] with promising evidence indicating that these digital interventions can significantly improve clinical outcomes [14].

A number of mHealth applications have been developed for individuals with OA although to date, most have tended to focus on mobile assessment, joint measurement, and motion monitoring tools [15] with very few focusing on OA management [16, 17]. This is despite other digital interventions demonstrating benefits to physical function and OA related symptoms [18, 19]. Those mHealth interventions focusing on lifestyle management have been developed to improve exercise adherence using a smartphone based exercise programme [17] or by tracking the patients steps (from a physical activity tracker), pain and mood whilst also delivering motivational messaging [16]. However, reports suggest that the long-term maintenance of physical activity (PA) in knee OA (KOA) patients without the ongoing support of a healthcare professional requires personalised PA guidance, education, and social support [20–22].

The intelligent knee osteoarthritis lifestyle app (iKOALA) has been co-developed with target users (i.e., people with KOA) to extend the support for PA and musculoskeletal (MSK) health beyond short-term, structured rehabilitation through personalised PA guidance, education, and social support [23]. This study aimed to assess the usability of the iKOALA intervention over a 12-week duration and to assess its impact on indices of musculoskeletal health, symptoms, and PA in a broad range of individuals with diagnosed knee KOA.

## Methods

### Participants

Study participants were recruited from across the UK with the assistance of PA champions at Versus Arthritis, a registered UK charity, as well as through social media advertising (Facebook, LinkedIn and Twitter) between January-August 2022. Recruitment was targeted at anyone diagnosed with KOA. A total number of 51 participants were consented to participate in this study to allow for anticipated attrition during the trial. Thirty-eight of these completed the full 12-week trial. Participants agreed to participate in the study after reading the electronic participant information sheet by providing their informed consent via an electronic form (onlinesurveys.ac.uk). The study was approved by the Department for Health Research Ethics Committee (REACH) at the University of Bath (Ref: EP 20/21–084). Table 1 describes the participant demographics involved in the study.

**Table 1:** Semi-structured interview questions

**1**	**General**
1a 1b	How would you describe your experience with the iKOALA App? Have you ever used a website or app’s that have a similar purpose to iKOALA? (i.e. provides information, physical activity advice or social support).
**2**	**Features**
2a	Can you tell me what the most useful features of the iKOALA app were for you?
2b	Was there anything you didn’t use or didn’t find useful?
2c	Can you tell me anything about the app that you thought was particularly good?
2d	Can you tell me anything about the app that you were less keen on?
2e	Is there anything else you would like to see included in the app?
**3**	**Usability**
3a	Overall, how easy did you find it to learn how to use the app?
3b	Did you use the information included in help function to help you understand iKOALA?
3c	Is there anything about how the app works in general or the ease of navigating the app that you would like to comment on?
3d	If you were to continue using iKOALA, how often do you think you would use it?
**4**	**Potential for use**
4a	Do you think you would continue using this app and if so, what would you use it for mostly?
4b	Is there anything you think would make you more likely to use iKOALA more frequently?
4c	Is there anything else you would like to add?

### Sample size

We estimated the sample size required to detect a moderate effect size (*d* = 0.5) on index knee pain from pre- to post-intervention, assuming a power of 0.8 and alpha probability of 0.05. The minimum required sample size was estimated at 34 participants. Our sample size for the qualitative interviews sub-sample was guided by the concept of information power where, given the relative homogeneity of the target population and the topic of focus, namely to learn about the intervention experiences, we determined a target sample of 10–12 (25–30% of the total sample) to be sufficient and practicable in the current study [24].

### Inclusion criteria

To be eligible to take part in the study, participants had to meet the following criteria:

- Been diagnosed radiographically or clinically with knee osteoarthritis by a clinician.
- Not have had any other condition, or a treatment related to another condition that prevented them from participating in PA.
- Not currently receiving regular (i.e., weekly) treatment from a physiotherapist or other healthcare practitioner related to their KOA.
- The ability to use an Android smartphone during the study (with operating system version 8 at a minimum) and be willing to download an app onto their device.
- A willingness to wear a wrist worn activity tracker during waking hours throughout the trial.

Each potential participant completed an online eligibility survey (onlinesurveys.ac.uk) to self-report their suitability for the trial. There were no restrictions around the participant’s current level of activity or severity of symptoms for inclusion in the study.

iKOALA.

The iKOALA is a mobile health application that has been developed to extend the support for PA and MSK health, beyond short-term structured rehabilitation, using personalised PA guidance, education, and social support. The development of iKOALA [23] involved a systematic iterative and interconnected development process comprising intervention ‘planning’ and ‘optimisation’ informed by the person-based approach (PBA) framework for the development of digital health interventions [25]. Specifically, iKOALA offers support for PA and MSK through:

1. Personalised PA guidance—Users are required to answer a series of questions with nominal answers about their PA preferences such as how active are you?/do you experience falls?/would you like to perform an activity as part of a group? Based on these answers, a library of activities is modified to suggest suitable activities for the individual. Users are then encouraged to add as many of these activities to their personalised activity plans to meet their PA goals which could be modified at any point by the user. Users are also able to connect a Fitbit (Fitbit, USA) or Google Fit (Google, USA) physical activity tracker displaying physical activity data in the iKOALA app.
2. Education—Users can access a range of educational material relating to KOA including what is KOA?/how will it affect me?/weight management/medication/reducing the strain on your knee etc.
3. Social support - Users can access activity-specific chat forums (e.g., walking, cycling etc.) within the app to connect with other iKOALA users, share their experiences and provide social support.

**Fig. 1:**
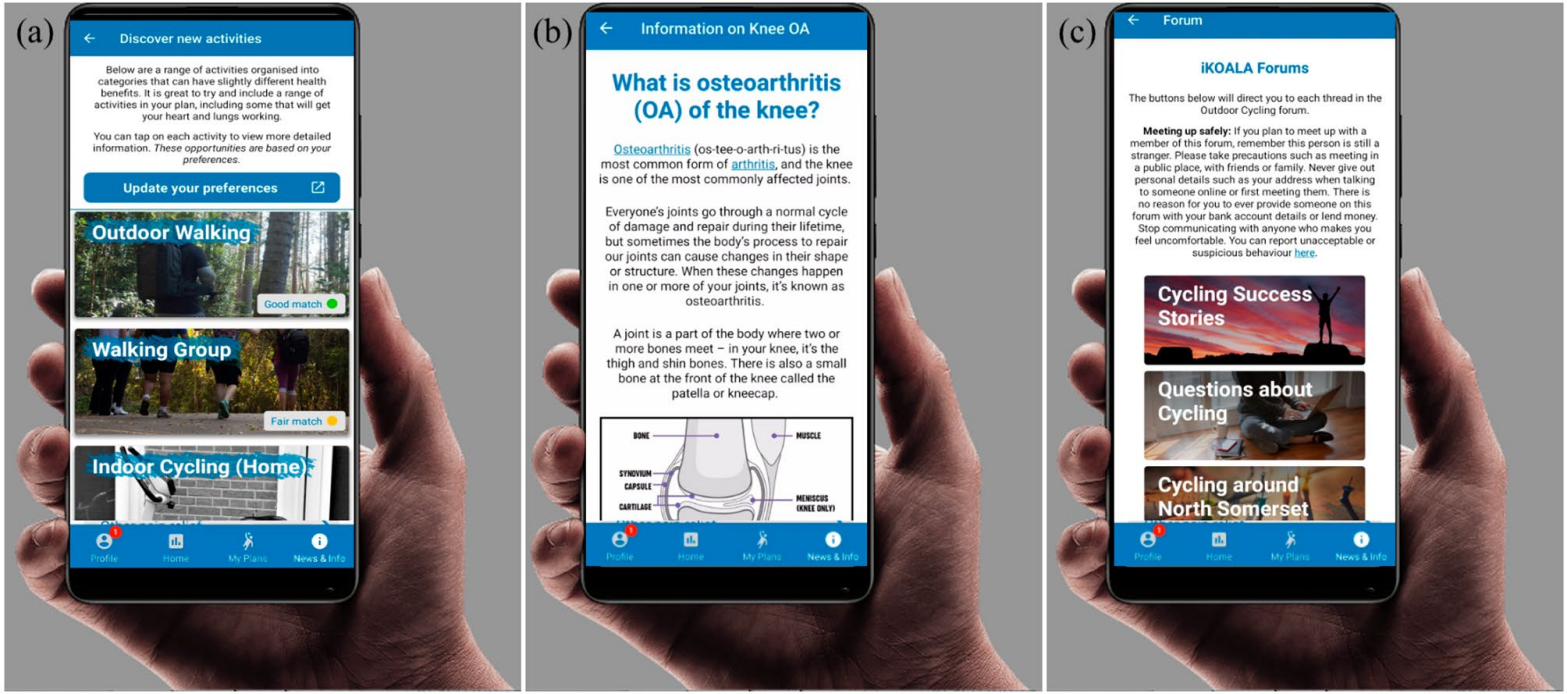
User view of the basic functions of iKOALA including **a** personalised PA guidance; **b** education material; **c** forums for social support

Figure 1 provides a user view of the basic functions of the iKOALA App including how to select suitable physical activities (a), the educational material (b) as well as the iKOALA forums (c).

### Protocol

This study used a mixed methods single group pre-post-test design to evaluate the effectiveness and usability of iKOALA [26]. The trial required study participants to wear a wrist worn PA tracker (Fitbit Inspire 2, Fitbit, USA) and to engage in a 12-week personalised PA plan via the iKOALA intervention. The trial was conducted remotely without the need for participants to attend any appointments or meetings at a specific venue. All communication between study participant and the researchers was conducted by telephone and email as and when required. After providing consent, participants were sent a PA tracker through the mail. Participants were instructed to set the activity tracker and associated app up on their smartphone with support from a researcher if required. Following this, participants were sent a link to download the iKOALA application with written instructions on how to install and set it up on their personal device. Once complete participants were instructed to link the Fitbit and iKOALA apps via the in-app iKOALA settings.

Participants were then asked to complete the iKOALA PA questionnaire to determine their appropriate level of activity. They then personalised their activity plan by choosing aerobic and strength based activities from the iKOALA library of exercises, and a start date was agreed with each participant. Following this, participants were asked to access the help tutorials in the app to help familiarise themselves with the intervention. Participants were encouraged to log any physical activity sessions completed and to use the iKOALA intervention to monitor their PA and symptoms. During the trial, a researcher checked in with each participant at the end of week 1, 4 and 8 to ensure there were no issues with the technology. Other than this, participants received no other regular communication from the researcher.

### Outcome measures

In the week prior to starting the trial, participants were asked to complete 2 questionnaires to assess their MSK symptoms within the iKOALA app. The MSK-HQ musculoskeletal outcome measure questionnaire [27] was used to assess chronic pain, symptoms (aches and stiffness) and quality of life. The MSK-HQ is a brief questionnaire that allows people with musculoskeletal conditions to report their symptoms and quality of life in a standardised way [28] and has been shown to a valid tool for the assessment of MSK pain [27, 29] where a change in score of 5.5 or greater is considered a minimal clinically important difference [30]. A bespoke iKOALA acute symptoms questionnaire was used to assess present levels of confidence, fatigue, mood, pain, sleep quality and ability to walk based on a 5-point Likert scale (1—not at all, 2 = slightly, 3—moderately, 4—severely, 5—extremely) [31]. The same questionnaires were completed in the week following the end of the 12-week trial.

The PA data collected included steps, sedentary minutes, light active, fairly active and very active minutes per day. The data was retrieved from participants physical activity tracker accounts following completion of the trial. At least 4 valid days per week with > 80% of data for that 24-h period, including at least one weekend day, were required for the data to be included in the analysis. In the week following the completion of the trial, participants were asked to complete a 10-item system usability scale questionnaire to evaluate the subjective usability of the intervention [31, 32] and were also invited to take part in a semi-structured one-to-one interview by video conference to gain their views on iKOALA. The interviews were conducted by a single researcher (RS) with oversight and training provided by an experienced qualitative researcher (MW). Fourteen questions broken down into 4 main themes (general experiences with the app, features, usability, and potential for use) were posed to participants and conducted within 7 days of completing the trial (see Table 1). The interviews were audio or video recorded, with prior consent and transcribed verbatim. Finally, iKOALA usage data was also extracted from the mobile application development platform (Firebase, USA) to understand the time users spent on the app throughout the trial.

### Analysis

All quantitative statistical analyses were completed using IBM SPSS version 28 (IBM, New York, USA). Descriptive data were analysed as frequencies and percentages. A one-way repeated measures analysis of variance (ANOVA) was used to analyse differences in group mean PA data between weeks 1, 6 and 12 during the trial. A dependent samples t-test was used to analyse the group mean differences in pre- and post-trial data for the MSK-HQ and iKOALA symptom scores. A long-run error rate of 5% (alpha = 0.05) was set *a priori* such that *p <* .05 was deemed statistically significant. Exact *p*-values are given unless *p* < .001. Cohen’s *d* was used to calculate standardised effect sizes and the meaningfulness of a statistically significant result where an effect size of 0.2 was considered small, 0.5 moderate and 0.8 or above large [33]. Qualitative interviews were analysed by 1 researcher (RS) using thematic analysis to identify, analyse and report repeated patterns within the data using a deductive and latent approach. This was guided by the process described by Braun and Clarke (2006) by becoming familiar with the data, creating initial codes, searching for themes, reviewing the themes, defining and naming the themes and finally interpreting them within the context of the project [34]. NVivo 7 (http://www.qrsinternational.com/) was used to organise and manage the qualitative data for the thematic analysis. All participant data were coded for confidentiality purposes.

## Results

We recruited thirty-eight (33 female) participants who had a mean age of 58 (± 9) years and were diagnosed with OA in one (26%) or both (74%) knees. Table 2 has demographic information for participants recruited in the study.

**Table 2:** Demographic details of participants included in the study

	Total participants (*n* = 38)
**Gender**	
Male	5 (13%)
Female	33 (87%)
**Age**	
30–39 years	1 (3%)
40–49 years	6 (16%)
50–59 years	16 (42%)
60–69 years	12 (31%)
70–79 years	3 (8%)
**Ethnicity**	
White - British	36 (95%)
Asian - Chinese	2 (5%)
**Knees affected by OA**	
One	10 (26%)
Two	28 (74%)
**Suffered with knee pain**	
Less than 1 year	1 (3%)
1–4 years	12 (31%)
5–9 years	13 (34%)
10–19 years	4 (11%)
20 + years	8 (21%)
**Highest level of education**	
Secondary school qualifications, or equivalent (aged 16+)	3 (8%)
Further education, or equivalent (aged 18+)	9 (24%)
Foundation degree or equivalent	5 (13%)
Undergraduate degree equivalent	14 (37%)
Master’s degree or equivalent	5 (13%)
PhD or equivalent	2 (5%)
**Employment status**	
Full-time employed	11 (29%)
Part-time employed	8 (21%)
Unemployed	4 (11%)
Retired	15 (39%)

### Physical activity data

PA data for 27 participants was retrieved following the completion of the trial. The group mean (± SD) PA data are presented in Table 3 indicating that physical activity levels remained relatively high and were consistent over the 12 weeks.

**Table 3:** Average daily group mean (± SD) PA data

Variable (Mean ± SD)	Week 1	Week 6	Week 12	Difference
Steps	9102 ± 3514	9576 ± 4214	9596 ± 3694	*F* = 1.863; *p* = .184
Distance (miles)	6.10 ± 2.52	6.40 ± 2.90	6.43 ± 2.67	*F* = 1.584; *p* = .403
Sedentary minutes	778 ± 187	806 ± 184	792 ± 219	*F* = 1.190; *p* = .312
Light active minutes	229 ± 66.9	241 ± 75.0	239 ± 68.7	*F* = 1.683; *p* = .196
Fairly active minutes	23.6 ± 13.0	24.1 ± 18.0	21.2 ± 18.6	*F* = 0.619; *p* = .543
Very active minutes	31.0 ± 24.9	29.5 ± 31.8	30.3 ± 28.1	*F* = 0.137; *p* = .872

### MSK-HQ

**Fig. 2:**
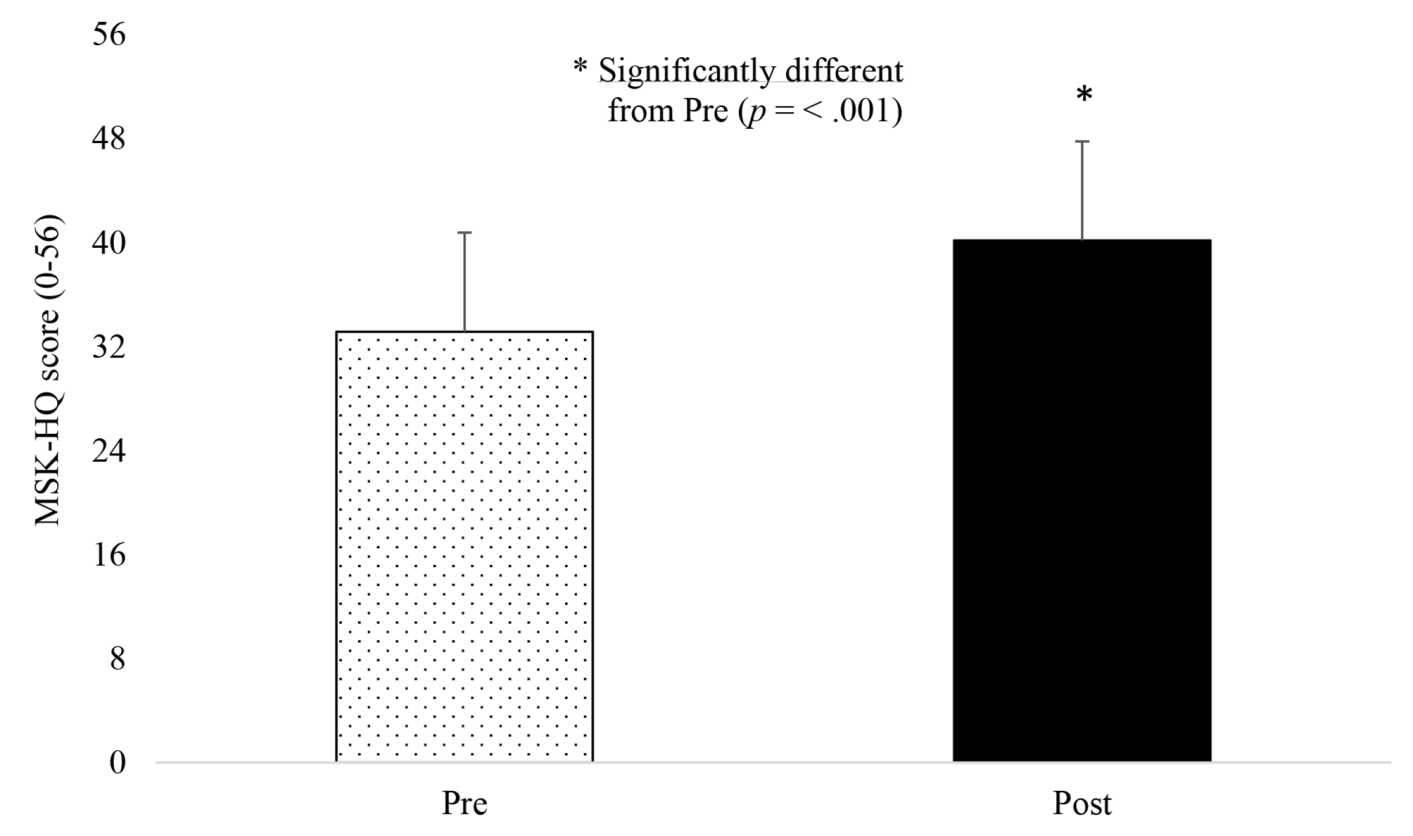
Group mean (± SD) MSK-HQ scores pre and post user trial

Thirty-four of the 38 participants completed the pre- and post-trial MSK-HQ questionnaire. Group mean (± SD) MSK-HQ scores increased over the trial from 33.1 (± 7.6) at pre to 40.2 (± 7.6) post-trial (Fig. 2) which was significantly different and with a large effect size (*t*(33) = 7.68, *p* < .001, *d* = 1.32, 95% CI [-8.89, -5.16]). This increase of 7.1 on the MSK-HQ score is greater than the minimum clinically important difference of 5.5 demonstrating a meaningful clinical improvement in overall MSK health.

### Symptoms

**Fig. 3:**
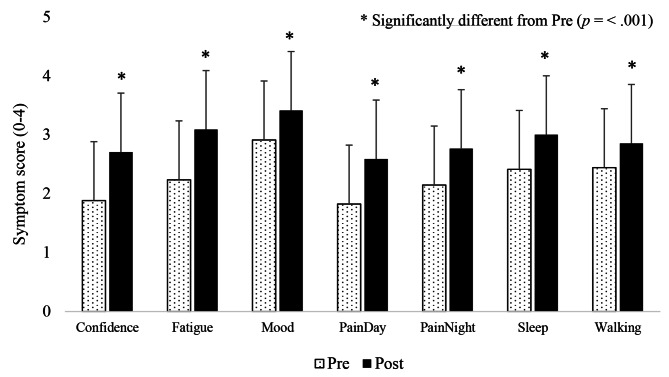
Group mean (± SD) symptom scores pre- and post-user trial

Thirty-four of the 38 participants completed the pre- and post-trial symptoms questionnaire. Figure 3 shows the group mean iKOALA symptoms scores pre and post-trial. For all variables, symptoms improved significantly and with moderate to large effect sizes for, mood (*t*(33) = -3.70, *p* < .001, *d* = 0.63, 95% CI [-0.77, -0.22]), night pain (*t*(33) = -4.41, *p* < .001, *d* = 0.76, 95% CI [-0.90, 0.33]), sleep quality (*t*(33) = -3.85, *p* < .001, *d* = 0.66, 95% CI [-0.90, -0.28]) and walking ability (*t*(33) = -3.94, *p* < .001, *d* = 0.68, 95% CI [-0.62, -0.20]) and large effect sizes for confidence (*t*(33) = -6.34, *p* < .001, *d* = 1.09, 95% CI [-1.09, -0.56]), fatigue (*t*(33) = -7.57, *p* < .001, *d* = 1.30, 95% CI [-1.08, -0.56]) and day pain (*t*(33) = -6.02, *p* < .001, *d* = 1.03, 95% CI [-1.02, -0.51]).

### App usage

**Fig. 4:**
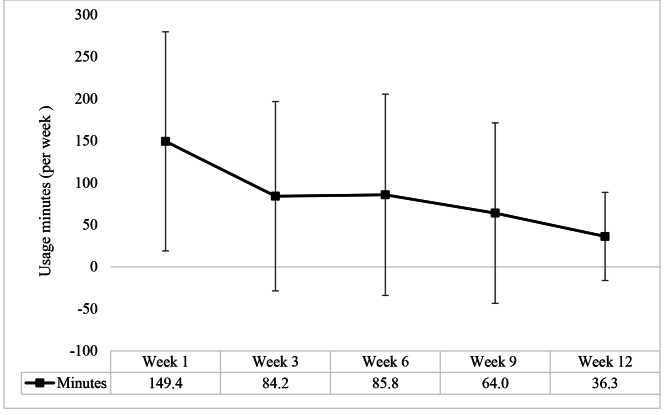
Group mean (± SD) usage of iKOALA per week (mins)

iKOALA app usage data was collected for all 38 participants. Figure 4 shows the weekly mean usage in minutes per week which reduced from 149 min in week 1 to 86 min in week 6 and 36 min in week 12.

### System usability scale

Thirty-two of the 38 participants completed the post-trial system usability questionnaire. Table 4 presents the most common (modal value) response from the participants’ subjective opinions of the iKOALA app indicating broadly positive feedback of the intervention.

**Table 4:** Group mode and mean (± SD) system usability scale scores post-trial

Question	Modal value*	Mean (SD)
1. I think that I would like to use this app frequently	4	4 (1)
2. I found this app unnecessarily complex	2	2 (1)
3. I thought this app was easy to use	4	4 (1)
4. I think that I would need assistance to be able to use this app	1	2 (1)
5. I found the various functions in this app were well integrated	4	4 (1)
6. I thought there was too much inconsistency in this app	2	2 (1)
7. I would imagine that most people would learn to use this app very quickly	4	4 (1)
8. I found this app very cumbersome/awkward to use	2	2 (1)
9. I felt very confident using this app	4	4 (1)
10. I needed to learn a lot of things before I could get going with this app	2	2 (1)

* Values: 1 = strongly disagree, 2 = disagree, 3 = neutral; 4 = agree, 5 = strongly agree

### Semi-structured interviews

Ten participants completed one-to-one semi-structured interviews which lasted 24 min on average (range 20–30 min) and provided both positive and negative feedback based on a series of semi-structured open-ended questions to understand the advantages and disadvantages of the iKOALA intervention.

### Advantages

The advantages that participants identified were categorised into 2 main themes: [1] benefits of using the app and [2] useful features within the app. In relation to the benefits, participants identified that the iKOALA intervention motivated them to maintain their chosen activity plan.

*“By following the iKOALA thing, it’s made me do them [exercises] in a way. Obviously, it doesn’t make me, but it encourages me to sit down and think right, I’ll do that, that and that.” (P18, female)*.

*“Well, the only thing I could say is, to be honest, if I hadn’t had the app, I wouldn’t have started doing more strengthening exercises.” (P48, female)*.

In relation to the useful features, participants identified several elements within the app that supported them in maintaining their PA plan during the trial. Firstly, having the relevant information within an app on their smartphones made the information both accessible and convenient.

*“It was easily accessible, whereas with some [apps], you’ve got to go into this and go into that to find these exercises, whereas with the iKOALA, I can just press a button, and they’re there.” (P18, female)*.

Participants also felt that having feedback on their current activity levels and current symptoms enabled them to ensure they were meeting their PA goals within sensible limits.

*“The bit that tells you how sedentary and how active you had been, was very good I found with the colours because you could just look at it and instantly see by the colours that - oh yeah, I’m doing well on that.” (P48, female)*.

*“[I was] looking at the graphs sometimes and going, alright then, that caused more pain but then it took time for that pain to actually calm back down again.” (P19, female)*.

### Disadvantages

The disadvantages associated with the app were focused on either technical issues or the inability to personalise some aspects of the content. The technical problems related to participants receiving repeated notifications for a period of the trial which was resolved approximately 4 weeks after first being reported.

*“Well, of course the notifications weren’t working, but they seem to be working now. Especially when you record how you’re feeling after the exercise, then you set a reminder to ask you the following day.” (P24, female)*.

In relation to being able to personalise content, when selecting activities from the iKOALA library of exercises, some participants would have liked to have been able to create their own specific activities and add it to their personalised PA plan.

*“It would be useful if you had an ‘other’[box]. So, if somebody was doing something that you hadn’t thought of, they could put it down and describe it, and then that would come up next time. And you might even think about whether to offer it to other people as well.”(P24, female)*.

*“In the same way as you do with the health boosts, you could add your own [exercises], really. So maybe you could add exercises that your own physio had given you.” (P19, female)*.

## Discussion

This study assessed the usability of the iKOALA mHealth intervention over a 12-week duration and its impact on indices of musculoskeletal health, symptoms, and PA in a broad range of individuals with diagnosed KOA. After completing the trial, participants reported significant and meaningful improvements in acute MSK symptoms and quality of life measures including less pain which is consistent with findings from the other mHealth KOA lifestyle applications [16, 17]. Unfortunately, a minimum important change has not been established for the iKOALA MSK symptom scores yet, so this could not be reported. The study also reported a statistically significant improvement in the combined MSK-HQ score to assess chronic joint, back, neck, bone and muscle symptoms. An improvement of 7.1 on the MSK-HQ score is in excess of the minimum important change of 5.5 demonstrating a meaningful improvement in overall MSK health [30]. Physical activity was also maintained at on average around 9000–9500 steps per day throughout the user trial. We are uncertain whether this was simply an active sample of KOA users or that they increased their activity levels immediately upon using the app. A larger, randomised controlled trial including baseline PA data would be a useful next step to evaluate the effectiveness of iKOALA when compared to standard care.

In the present study, participants also reported a statistically significant improvement in walking ability and this finding was supported by the qualitative interview data. During the interviews, several participants referred to the iKOALA intervention as being a motivating and supportive influence to maintaining their PA goals. The quantitative data also indicated that after starting the personalised physical activity programme, there was no significant changes in group mean PA for steps, sedentary, light active, fairly active or very active minutes between weeks 1, 6 and 12. These results could be promising because it has been previously identified that without regular contact with a healthcare professional, adherence to exercise plans often decline leading to poorer health outcomes, however this was not the case in this study [22, 35]. However, our week 1 intervention data demonstrated high levels of activity amongst the participants in the study, and an objective evaluation of PA prior to the trial would be useful step in clarifying the impact of iKOALA on usual PA levels.

Other studies have also reported improved adherence to an exercise programme using mHealth technologies. Alasfour & Almarwani (2022) found that participants that used the app based activity programme had significantly better adherence rates when compared to those assigned to the paper based exercise programme [17]. The convenience of using a smartphone to store a physical activity plan was one of the main reasons attributed to the higher adherence rates, and this is similar to the findings from the qualitative feedback in this study. Skrepnik et al. (2017) also reported improvements in PA when combining a mHealth app with a PA tracker [16]. Unfortunately, baseline PA was not collected in this study to determine if PA improved from baseline measures.

The system usability scale and semi structured interviews provided useful information on the usability of the iKOALA intervention. Whilst a small number of participants experienced technical issues during the trial, on the whole participants predominantly reported that the app was easy to use and that the various functions in the app were well integrated. This is a substantial improvement in the subjective usability when compared to a previous version assessed during the development phase [23] suggesting that subsequent iterations based on user feedback has been successful in improving usability and acceptability.

Whilst participants also reported that they would like to use the app frequently, we did see a drop off in weekly average app usage time in this study. Considering none of the participants had used the app prior to starting the trial, it is unsurprising that the largest drop in usage was between weeks one and three. This is because participants were asked to make themselves familiar with the app by viewing the help sections as well as getting comfortable with entering and reviewing their data. However, it is unclear why, after a plateau in usage between weeks three and six, app usage continued to decline from weeks 6 to 12, although it is acknowledged that the disengagement with mHealth apps is not uncommon [36, 37]. Interestingly, despite a reduction in app usage, we did not see similar reductions in PA. One speculative explanation for this may be that by week 6, a new exercise routine had been adopted and engagement with the app had peaked, however, it would be useful to understand what happens to both app usage and adherence to the PA plan over a longer duration. Additionally, because of the known attrition in mHealth app usage, it may be important to embed a number of strategies to encourage ongoing engagement including gamification [38].

While the results from the participants are promising, there are several limitations associated with this study and thus they should be treated as exploratory rather than definitive. Firstly, because the costs associated with developing a cross platform (iOS and Android platform) was cost prohibitive at this stage of the development process only android users were able to take part in the study which hindered our recruitment opportunities. Secondly, because the benefits of any self-management mHealth application lies on long term engagement, it is not known what may happen to app usage and ultimately engagement in the PA programme. As such a longer trial would provide a better understanding in this regard and help determine the longer term health and wellbeing impact of using iKOALA. Furthermore, this study had no control group to help us isolate the impact of the iKOALA app, therefore a randomised controlled trial in a real-world setting would be beneficial. This would provide further opportunity to validate the secondary outcome measures of the acute iKOALA MSK symptom scores. Additionally, the sample size was relatively small and with relatively few male participants so lacks generalisability to male, non-white, low SES people [39]. Whilst we are uncertain as to why so few male participants volunteered for the study, this is something that we would proactively consider if undertaking a larger randomised control trial in the future. Finally, baseline PA levels would have been useful in order to understand the immediate impact of the iKOALA app on objective measures of PA.

## Conclusions

After engaging with the iKOALA intervention for a period of 12 weeks, participants reported significant and meaningful improvements in both acute and chronic MSK symptoms and quality of life measures. These results therefore suggest that the combination of personalised PA guidance, education, and social support in a mHealth intervention can support individuals in self-managing KOA, which may be useful to individuals that are unable to access face to face support because of financial, geographical, or other access reasons. These results may be useful to clinicians and researchers where digital interventions are being considered to support patients managing their KOA symptoms.

## Data Availability

The datasets generated and/or analysed during the current study are not publicly available but are available from the corresponding author on reasonable request.

## Abbreviations

iKOALA: Intelligent knee osteoarthritis lifestyle app
KOA: Knee osteoarthritis
mHealth: Mobile health
MSK: Musculoskeletal
OA: Osteoarthritis
PA: Physical activity
REACH: Department for Health Research Ethics Committee
